# Pharmacotherapy for acute mania and disconcordance with treatment guidelines: bipolar mania pathway survey (BIPAS) in mainland China

**DOI:** 10.1186/1471-244X-14-167

**Published:** 2014-06-05

**Authors:** Zuowei Wang, Keming Gao, Wu Hong, Mengjuan Xing, Zhiguo Wu, Jun Chen, Chen Zhang, Chengmei Yuan, Jia Huang, Daihui Peng, Yong Wang, Weihong Lu, Zhenghui Yi, Xin Yu, Jingping Zhao, Yiru Fang

**Affiliations:** 1 Division of Mood Disorders, Shanghai Mental Health Center, Shanghai Jiao Tong University School of Medicine, Shanghai 200030, P. R. China; 2 Division of Mood Disorders, Hongkou District Mental Health Center of Shanghai, Shanghai 200083, P. R. China; 3 Mood and Anxiety Clinic in the Mood Disorders Program, Department of Psychiatry, University Hospitals Case Medical Center/Case Western Reserve University School of Medicine, Cleveland, OH 44106, USA; 4 Institute of Mental Health, Peking University, Beijing 100191, P. R. China; 5 Mental Health Institute,The Second Xiangya Hospital of Central South University, Changsha, Hunan 410011, P. R. China

**Keywords:** Bipolar disorder, Mania, Pharmacotherapy, Treatment, Guidelines

## Abstract

**Background:**

With the recent attention to evidence-based medicine in psychiatry, a number of treatment guidelines for bipolar disorders have been published. This survey investigated prescribing patterns and predictors for guideline disconcordance in the acute treatment of a manic and mixed episode across mainland China.

**Methods:**

The pharmacological treatments of 2828 patients with a recent hypomanic/manic episode or mixed state were examined. Guidelines disconcordance was determined by comparing the medication(s) patients were prescribed with the recommendation(s) in the guidelines of the Canadian Network for Mood and Anxiety Treatments.

**Results:**

The most common pattern of pharmacological treatments for an acute manic or mixed episode was a mood stabilizer plus an atypical antipsychotic (n = 1345, 47.6%), and the rate of guideline-disconcordant treatments was 11.1%. The patients who were treated in general hospitals were more likely to receive guideline-disconcordant treatments than those who were treated in psychiatric hospitals, with an OR of 1.84 (95% CI 1.44-2.36). Similarly, the patients with a mixed episode at study entry were more likely to receive guideline-disconcordant treatments than those with a manic episode, with an OR of 1.69 (95% CI 1.22-2.35). In contrast, the patients with a longer duration of disease (>5 years) were less likely to receive guideline-disconcordant treatments than those with a short duration, with an OR of 0.47 (95% CI 0.36-0.60).

**Conclusions:**

In mainland China, the disconcordance with treatment guidelines for a most recent acute manic or mixed episode was modest under naturalistic conditions. The higher risk for disconcordance in general hospitals than in psychiatric hospitals suggests that special education based on treatment guidelines to practitioners in general hospitals is necessary in order to reduce the risk for disconcordant treatments.

## Background

There is growing recognition that bipolar disorder (BPD) is common, with bipolar disorder type I (BP-I) and type II (BP-II) affecting about 2% of the world’s population and subthreshold forms of the disorder affecting another 2% [1,2]. BPD is responsible for the loss of disability-adjusted life years (DALYs) more than all forms of cancers or major neurological conditions, primarily because of its early onset and chronicity across the life span [2]. The recurrent nature of manic and depressive episodes often leads to high direct and indirect health care costs [3,4]. Moreover, BPD is also a leading cause of premature mortality due to suicide and comorbid medical conditions such as diabetes and cardiovascular diseases [5,6].

There is evidence that greater provider adherence to treatment guideline recommendations was associated with a greater reduction in symptoms and greater improvement in the outcome of diseases [7,8]. For this reason, a number of treatment guidelines have emerged to aid clinicians to make clinical decisions for the treatment of patients with BPD. These practice guidelines include, but not limited to, the guideline of the American Psychiatric Association [9], the British Psychological Society [10], the Chinese Medical Association [11], the World Federation of Societies of Biological Psychiatry [12-14], the Health Ministry of Singapore [15], and the Canadian Network for Mood and Anxiety Treatments (CANMAT) and the International Society for Bipolar Disorders (ISBD) [16].

Although the timeline of these guidelines has spread more than a decade, recent advances in pharmacological treatment for patients with acute mania remain quite modest [16,17], lithium, valproate/divalproex, carbamazepine, and antipsychotics have been the cornerstones for the acute treatment of mania for decades. More importantly, treatment-disconcordance in acute mania has continuously been reported. In a previous survey with 224 mood episodes in 84 outpatients with BPD from the UK, the rate of guidelines concordance was 81% for manic episode [18]. A large-scale and prospective study in 964 outpatients with BPD from the US reported that the guideline-concordant treatments for hypomanic/manic and mixed episode(s) were 82% [19]. However, a more recent study in 113 individuals with BPD who were referred to a Mental Health Centre from the community found that the rate of guideline-concordant treatment based on CANMAT& ISBD guidelines for hypomania was only 32% [20].

There was no update on treatment-guidelines for bipolar disorder in mainland China since 2007. Until now there has never been a study on the guidelines concordance for the treatment of BPD in China. This report is to present the data of a national survey (Bipolar Mania Pathway Survey, BIPAS) on the acute treatment of a recent manic or mixed episode in routine clinical practice in mainland China.

## Methods

### Participants

The BIPAS study was conducted in 26 sites (15 psychiatric hospitals and 11 psychiatric departments of general hospitals) across mainland China from the November of 2012 to the January of 2013. All procedures were reviewed and approved by the institutional review boards of Shanghai Mental Health Center and other participating institutions. Written informed consents were obtained from each participant before any study-related procedure was performed. Diagnosis of bipolar disorder was ascertained with *ICD-10* (International Classification of Disease – 10 Edition) diagnostic criteria for BPD by a review of medical records. Both inpatients and outpatients who presented with an acute manic/mixed episode or in remission were consecutively screened for this study without consideration of age limitation or other exclusion criteria.

### Clinical assessments

All patients who were enrolled in the study received a single-round survey. During the screening visit, variables including gender, hospital category, mood state at first onset and at study entry, current co-morbidity with mental disorders and/or physical disorders, family history of a mental disorder(s), the age at study entry and at first onset, the duration of BPD, and the number of episodes in past year were obtained from patients, their family members, and medical records by research psychiatrists and research assistants. The prescribing information (name of psychotropic agents) for a recent manic/mixed episode was retrospectively obtained from patients’ medical records. The collected psychotropic agents included mood stabilizers, antipsychotics, antidepressants, Chinese medicine, and benzodiazepines for treating mental symptom. Medications for dealing with physical diseases or side effects related to psychotropic agents were not collected.

### Disconcordance determination

Recommendations for pharmacological treatment of acute mania by the CANMAT and ISBD guidelines [16] were used to determine a treatment(s) as guideline concordance or disconcordance. The use of these guidelines as “standard” was justified because they were commonly recommended during continuing medical education and clinical practice in mainland China. Among the available psychotropic agents for bipolar mania in clinical practice in mainland China, mood stabilizer (lithium and valproate) monotherapy and atypical antipsychotic monotherapy (risperidone, olanzapine, quetiapine, aripiprazole and ziprasidone) were considered as the first-line treatments; adjunctive therapy of atypical antipsychotics with lithium or valproate, and monotherapy of carbamazepine and haloperidol as the second-line treatments; and lithium plus valproate, and monotherapy of chlorpromazine and clozapine as the third-line treatments. In this survey, the pharmacological treatment with at least one agent or combination recommended in CANMAT and ISBD guidelines was considered as guidelines concordance (without a sequential consideration of treatment options as first-line agents, second-line agents, and third-line agents). Therefore, other treatment option(s) was considered as guidelines disconcordance.

### Statistical analysis

Demographics characteristics, clinical features and treatment options for a recent depressive episode were analyzed with descriptive statistics. A conditional logistic regression with stepwise method among variables was used to study independent predictors for guidelines disconcordance. All candidate variables entered the model were categorical variables. Continuous variables were divided into categories as their medians. Given the exploratory nature of the study, statistical significance was set at α = 0.05, two-tailed without adjustment for multiple comparisons.

The variables analyzed in the regression model included gender (male vs. female), hospital category (psychiatric vs. general), mood state at first onset (hypomanic/manic episode vs. depressive episode), mood state at study entry (hypomanic/manic episode vs. mixed episode), current co-morbidity with mental disorders (no vs. yes), current co-morbidity with physical illness (no vs. yes), a family history of mental disorder(s) (no vs. yes), the age at study entry (≤32 years vs. >32 years), the age at first onset (≤24 years vs. >24 years), the duration of disease (≤5 years vs. >5 years), and the number of episodes in past year (≤1 time vs. >1 time).

## Results

### Demographics and clinical characteristics

Totally, 3906 participants with BPD were screened for this survey. After excluding patients with a recent depressive episode, 2828 patients with a recent hypomanic, manic, or mixed episode were enrolled into the present study. The majority of patients had a recent hypomanic or manic episode and the first hypomanic or manic episode, and was treated in psychiatric hospitals (Table 1). Most patients did not have current mental or physical comorbidity, or a family history of mental disorders. The median age at the study entry was 32 years old. The median age at the onset of first mood episode was 24 years old. The median duration of BPD was 5 years. The median number of mood episodes in the past year was one episode (Table 1).

**Table 1:** Demographics and clinical characteristics of patients with acute manic and mixed episodes

**Characteristic**	**N**	**%**
Gender		
-Male	1468	51.9
-Female	1360	48.1
Hospital category		
-Psychiatric	1754	62.0
-General	1074	38.0
Mood state at study entry		
-Hypomanic/manic episode	2520	89.1
-Mixed episode	308	10.9
Mood state at first onset		
-Hypomanic/manic episode	1925	68.1
-Depressive episode	903	31.9
Current co-morbidity		
-Mental disorders	99	3.5
-Physical disorders	203	7.2
Family history of mental disorders	582	20.6
	Median	IQR
Age at study entry (years)	24.0	19.0-32.0
Age at first onset (years)	32.0	24.0-44.0
Duration of disease (years)	5.0	2.0-12.0
Number of episodes in past year	1.0	1.0-2.0

### Patterns of pharmacological treatment for acute manic and mixed episodes

As shown in Table 2, of the 2828 patients, 1653 (58.5%) patients received treatment with two-drug combination with a mood stabilizer plus an atypical antipsychotic as the most commonly prescribed treatment (n = 1345, 47.6%). The second most commonly prescribed treatment was monotherapy (n = 622, 22.0%), which included mood stabilizer monotherapy (n = 275, 9.7%), antipsychotic monotherapy (n = 245, 8.7%), antidepressant monotherapy (n = 55, 1.9%) and other agent monotherapy (n = 47, 1.7%). Meanwhile, 344 (12.2%) patients received the combination of three or four psychotropic agents (n = 344, 12.2%), and 209 (7.4%) patients did not receive any psychotropic agent (Table 2).

**Table 2:** Patterns of pharmacological treatments in patients with acute manic and mixed episodes

**Treatment patterns**	**N**	**%**
*No agent*	*209*	*7.4*
*Monotherapy*	*622*	*22.0*
MS	275	9.7
AP (AAP and TAP)	245	8.7
AD	55	1.9
Other agent	47	1.7
*Two drugs combination*	*1653*	*58.5*
MS + AAP	1345	47.6
Combination with at least one agent recommended in guidelines	305	10.8
No treatment following the guidelines	3	0.1
*Three or four drugs combination*	*344*	*12.2*
MS(s) + AAP(s)	73	2.6
Combination with at least one agent recommended in guidelines	271	9.6

*MS* mood stabilizers, *AP* antipsychotic, *AAP* atypical antipsychotic (risperidone, olanzapine, quetiapine, aripiprazole, and ziprasidone), *TAP* typical antipsychotic (chlorpromazine, clozapine and haloperidol), *AD* antidepressant, *Other agent* Chinese medicine, benzodiazepine and unknown.

### Rate of guideline-disconcordant treatments in acute manic and mixed episodes

As shown in Table 2, 314 (11.1%) patients did not take any agent recommended in CANMAT guidelines for acute mania/hypomania, which was considered as guidelines disconcordance. The most common treatment of guideline-disconcordance was non-pharmacological treatment (7.4%), antidepressant monotherapy (1.9%), and other agent monotherapy (1.7%). If the monotherapy of a mood stabilizer or an atypical antipsychotic as well as the combination therapy of a mood stabilizer(s) plus an atypical antipsychotic(s) were strictly considered to be guideline-concordant, the rate of guidelines disconcordance would increase to 31.5%.

### Predictors of guideline-disconcordant treatments in acute manic and mixed episodes

Among the 11 variables considered for logistic regression analysis, 3 of them including hospital category, mood state at study entry, and duration of disease remained in the model as independent risk factors for guidelines disconcordance. The patients who were treated in general hospitals were more likely to receive guideline-disconcordant treatment than patients treated in psychiatric hospitals with an OR of 1.84 (95% CI 1.44-2.36). Similarly, those patients with a mixed episode at study entry were also more likely to receive guideline-disconcordant treatment than their counterparts with a manic episode at study entry, with an OR of 1.69 (95% CI 1.22-2.35). In contrast, patients with a longer duration of BPD (>5 years) was less likely to receive guideline-disconcordant treatments than their counterparts with a shorter duration of equal or less than 5 years, with an OR of 0.47 (95% CI 0.36-0.60) (Table 3).

**Table 3:** Predictors for guideline-disconcordant treatments in patients with acute manic and mixed episodes

**Predictors**	**Wald *χ***^***2***^	***P-value***	**OR**	**95% CI**
General hospitals	23.89	0.000	1.84	1.44-2.36
Mixed episode at study entry	9.77	0.000	1.69	1.22-2.35
Duration of disease (>5 years)	33.24	0.000	0.47	0.36-0.60

## Discussion

To our knowledge, this is the first national survey to investigate the current practice of pharmacological treatments and guidelines concordance for an acute mania or mixed episode in mainland China. The inclusion of both inpatients and outpatients from both psychiatric hospitals and general hospitals increase the generalizability of this study. According to the CANMAT and ISBD guideline recommendations without considering sequenced treatment alternatives, the disconcordant rate with guidelines in the present study was 11.1% (Table 2).

The disconcordant rate with the guidelines in our survey was lower compared with that previously reported in a Systematic Treatment Enhancement Program for Bipolar Disorder (STEP-BD) study from United States [19], in which the disconcordant rate was about 18% for hypomanic/manic and mixed episodes. This discrepancy could be due to the followings. First, the STEP-BD study collapsed all published clinical practice guidelines at the time into a composite guideline as the “standard” for guideline concordance or disconcordance. Second, both sequenced treatment alternatives and therapeutic dosages were considered in the STEP-BD study. Third, our present survey was conducted in large-scale psychiatric and general hospitals across mainland China. If strictly followed the recommendations in the CANMAT and ISBD guidelines, the disconcordant rate could be much higher than 11.1%.

The probable factors predicting guidelines disconcordance could be both physician-specific and patient-specific. The practice type and experience of clinicians may be important factors affecting clinical decisions and guideline concordance [7,21]. Those practitioners who did not comply with guidelines might believe that those guidelines did not address particular features of their clinical patient populations [22]. As reported previously, only 3.4% patients with a mood disorder had ever seen a mental health professional, 4.9% had ever sought a non-mental health professional in mainland China [23]. The general practitioners in general hospitals usually prescribe medication(s) for patients with a mood disorder in mainland China. Because general practitioners have little or no training in mental health, they are unable (and often unwilling) to provide basic psychiatric services for those with a mood disorder [23], which could be one plausible explanation for the absence of psychotropic treatment (7.4%) and the inappropriate use of antidepressants (1.9%) in this survey.

The finding of a mixed episode at study entry associated with increased risk for guidelines-disconcordance is consistent with previous studies [18,24]. Mania (81%) was more likely to be managed in accordance with guidelines than depression (64%) or mixed episodes (62%) [18]. The divergence from guidelines was mostly noted for patients with hypomania, which was associated with inappropriate treatments with antidepressants, absence of psychotropic treatment, and/or inadequate dosage [20]. Since our study focused on the current episode and did not make a distinction between hypomania and mania, the previous diagnose(s) and the current subtype of bipolar disorders could not be verified. Therefore, the proportion of discorntant-treatments due to misdiagnosis or correct diagnosis with inappropriate treatments is difficult to be determined. In contrast, a longer duration of bipolar illness was associated with the decreased risk for disconcordant treatments, suggesting that long-term illness could provide more opportunities for clinician(s) to make a correct diagnosis and proper treatment.

The finding of a mood stabilizer(s) plus an antipsychotic(s) as the most commonly prescribed pattern (more than 50%) in our study is consistent with a previous report that polypharmacy for acute manic and mixed episodes was a common phenomenon [24]. Since our study was cross-sectional and retrospective, the role of the combination therapy during the course of acute episode was unknown. However, the validity of this practice is still debatable. Some evidence from both clinical practice and randomized controlled trials showed that the addition of antipsychotic(s) to lithium or valproate was superior to lithium or valproate alone, but there is not enough unambiguous evidence that supports the combination therapy of a mood stabilizer with an antipsychotic as a general first-line treatment [12]. Considering a higher frequency and/or greater severity of side effects associated with combination therapy relative to monotherapy, a mood stabilizer(s) plus an antipsychotic(s) should be reserved for severe mania or as a subsequent step for mild to moderate mania [12]. Similarly, Post et al. reported that complex medication regimens were required to achieve a sustained response when monotherapy was unsuccessful [25]. Thus delineating the clinical and biologic correlates of individual response to combination treatment could be one of clinical research priorities in the future [25].

Several limitations of this study should be considered. First, this study was carried out in 26 large-scale psychiatric and general hospitals across mainland China. They are all located in the provincial capital cities or municipalities and directly under the central governments’ control. The current practice for the treatment of patients with BPD in small and medium-sized cities, village or community was not included in this study. Second, this study was a cross-sectional and retrospective investigation based on medical records, which did not allow us to study the changes in the treatment regimes for patients with acute manic and mixed episodes. Some psychotropic agents could be the continuation from previous treatment regimes for previous mood episodes. Therefore, some agents might not be used specially for the recent manic and mixed episodes. Third, the studied demographics and clinical characteristics were most patient-specific factors. Physician-specific factors such as age, education level, and training experience were not available in the present study, which was reported to affect prescribing pattern and guideline concordance [7,21,22].

## Conclusions

In mainland China, the guideline-disconcordant treatments for patients with an acute hypomanic, manic or mixed episode were not common (11.1%) under naturalistic conditions. The absence of psychotropic treatment and the inappropriate use of antidepressants were the major factors for the disconcordance. The correlation between guidelines disconcordance and physician-specific (physicians from general hospitals) and patient-specific features (a mixed episode at study entry and a shorter duration of disease) factors suggest that in order to avoid improper psychotropic treatments for an acute mania/hypomania/mixed episode and to close the gap between treatment guidelines and clinical practice in mainland China, continuing education to practitioners, especially those in general hospitals, is necessary.

## Abbreviations

BIPAS: Bipolar mania pathway survey; BPD: Bipolar disorder; CANMAT: Canadian network for mood and anxiety treatments; ICD-10: International classification of disease–10 edition; ISBD: International Society for Bipolar Disorders; STEP-BD: Systematic treatment enhancement program for bipolar disorder.

## Competing interests

All authors declare that they have no competing interests.

## Authors’ contributions

ZW and KG contributed to design, analysis and interpretation of data and drafting the manuscript. XY, JZ and YF contributed to conception, design, and revising the manuscript. WH, MX, ZW, JC, CZ, CY, JH, DP, YW, WL, and ZY contributed the subjects’ enrollment and the clinical assessments. All authors have given final approval of the version to be published.

## Pre-publication history

The pre-publication history for this paper can be accessed here:

http://www.biomedcentral.com/1471-244X/14/167/prepub
